# A cadaveric assessment of percutaneous trigger finger release with 15° stab knife: its effectiveness and complications

**DOI:** 10.1186/s13018-021-02566-4

**Published:** 2021-07-03

**Authors:** Abbas Abdoli, Majid Asadian, Seyed Houssein Saeed Banadaky, Rabeah Sarram

**Affiliations:** 1 Department of Orthopedics, Shahid Sadoughi University of Medical Sciences, Yazd, Iran; 2 Shahid Sadoughi University of Medical Sciences, Yazd, Iran

**Keywords:** Trigger fingers, Percutaneous release, Success rate, A1 pulley, 15° stab knife

## Abstract

**Abstract:**

Percutaneous release of the A1 pulley has been introduced as a therapeutic approach for trigger fingers and is suggested as an effective and safe alternative, where conservative treatments fail. The aim of the current study was to determine if percutaneous release with a 15° stab knife can effectively result in acceptable efficacy and lower complication rate.

**Methods:**

In the present study, the percutaneous release of the A1 pulley was evaluated by percutaneous release using a 15° stab knife in 20 fresh-frozen cadaver hands (10 cadavers). One hundred fingers were finally included in the present study. The success rate of A1 pulley release as well as the complications of this method including digital vascular injury, A2 pulley injury, and superficial flexor tendon injury was evaluated, and finally, the data were analyzed by the SPSS software.

**Results:**

The results showed a success rate of 75% for A1 pulley release in four fingers, followed by eleven fingers (90%) and eighty-five fingers (100%). Therefore, the A1 pulley was found to be completely released in eighty-five fingers (100%). Overall, the mean of A1 pulley release for these fingers was determined as 97.9%, indicating that percutaneous trigger finger release can be an effective technique using a 15° stab knife. Furthermore, our findings revealed no significant difference in the amount of A1 pulley release in each of the fingers in the right and left hands. Additionally, 17 fingers developed superficial scrape in flexor tendons, while 83 fingers showed no flexor tendons injuries and no other injuries (i.e., vascular, digital nerve, and A2 pulley injuries).

**Conclusions:**

Percutaneous release of the A1 pulley using a 15° stab knife was contributed to acceptable efficacy and a relatively good safety in the cadaveric model.

## Introduction

The trigger finger (TF) or stenosing tenosynovitis has been defined as a condition caused by thickening of the flexor tendon sheath or its nodular thickening, resulting in a difference between flexor tendon diameters/retinacular sheath of flexor and the A1 pulley, contributing to the delayed and painful extension of the digit, pain, and disability [1, 2].

This implicates the A1 pulley sheath, A2 or A3 [3–5]; however, the primary pathology has been described to be thickened A1 pulley, which is associated with entrapment of the flexor tendon, leading to triggering sensation [6]. Although the exact cause of this disease is not clear, multiple factors such as repetitive finger movements (Repetitive strain injury)**,** local trauma, systemic conditions (e.g., diabetes and rheumatoid arthritis), stress, and degenerative force are associated with its occurrence [2, 7–9].

A trigger finger, with a 2 to 3% risk in the general population and up to 10% for patients suffering from diabetes mellitus, was found to be the most commonly occurred in the middle 5th to 6th decades of life, and thus is a common condition in adults [2, 10–12]. It is worth noting that the middle and ring fingers are described as the most common fingers implicated, and women (middle-aged women) are more affected by this condition as compared to men [2, 13].

Conservative treatment of trigger fingers has been described previously including anti-inflammatory drugs, corticosteroid injection for short-term symptom relief, and immobilization of the finger [8, 14]. Orthosis was found to be capable of immobilizing 1 finger joint for preventing triggering mechanism [14]. Percutaneous trigger finger release and surgery are other options for those who do not respond to consensus management. There are studies that indicated a lower success rate of conservative treatment including steroid injection in diabetic patients, splinting, and other non-operative modalities [15, 16], while percutaneous release has been introduced as a safe alternative, where conservative treatment fails because trigger digit was found to be effectively managed by percutaneous release [15]. Success rates of finger release have been reported to be between 84 and 100% by the mid-term follow-up [17–19].

However, surgical release is reported to be involved in complications such as persistence, recurrence infection, scar tenderness, digital nerve injury, flexion contracture, and bowstringing [20]. Therefore, the aim of this study was to evaluate the rate of A1 pulley release by the percutaneous trigger finger release (PTFR) method and its complications.

## Material and methods

This experimental study was carried out after approval of the Ethics Committee of Yazd University and Forensic Medicine Organization in Yazd. It should also be noted that written consent was obtained from the heirs. Inclusion criteria included 20 cadaver hands, ranging in age from 20 to 70 years, up to 36 h after their death. Exclusion criteria included a history of any injury to the hand and the presence of a scar or deep wound at the site of incision. Initially, A1 pulley release was performed on some corpses to enhance the surgeon’s skill, which was eventually excluded.

**Fig. 1:**
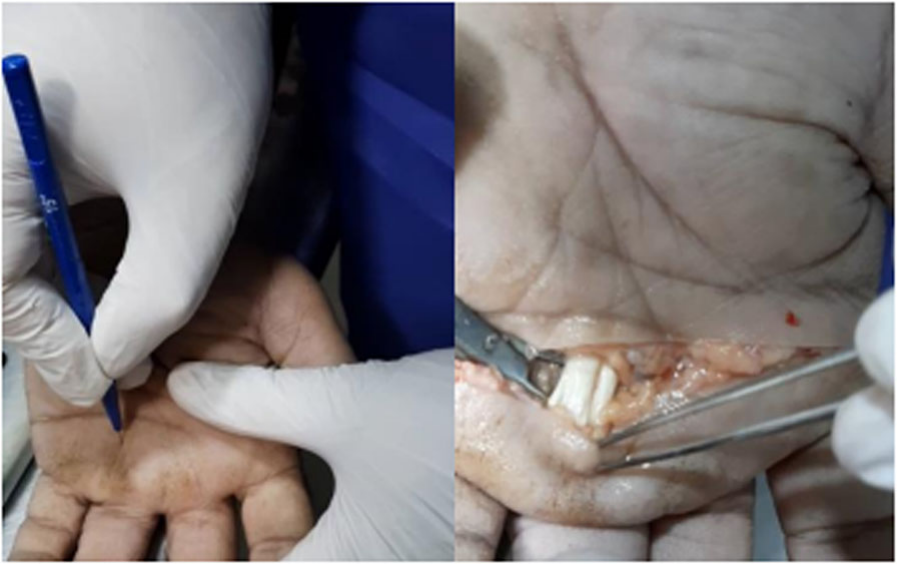
Percutaneous A1 pulley release

All cadaver hands underwent surgery by a surgeon, and post-operative photography was performed on the site (Fig. 1) and also evaluated by another surgeon’s colleague. The organs were validated 24 h prior to the procedure.

**Fig. 2:**
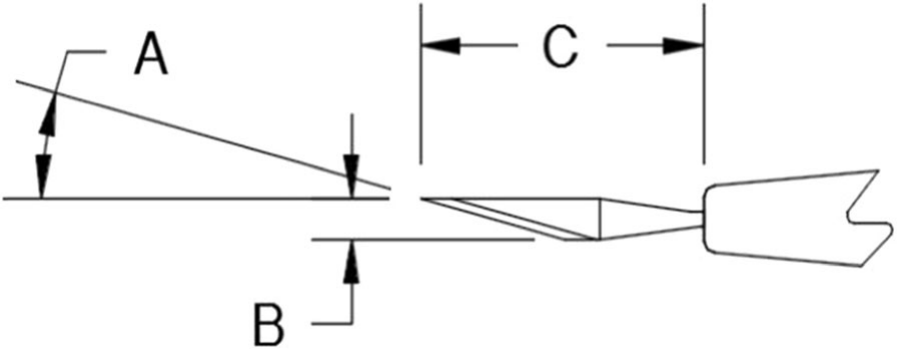
Fifteen-degree stab knife specification (A = 15°, B = 1.6 mm, C = 11 mm)

Percutaneous release of A1 pulley was performed according to the anatomical landmarks of each finger with a 15° stab knife (Fig. 2). The proximal and distal palmer creases were marked. All digits were in slight abduction position (except the middle finger). The midline of each finger was marked along the palm to the carpal tunnel through the palmar creases. The relative location of the A1 pulley was marked beneath a line drawn from the radial end of the proximal palmar crease to the ulnar end of the distal palmar crease at the marked midline level. The palpation technique using surface landmarks was performed along the marked A1 pulley combined with finger flexion and extension. The correct location of the A1 pulley can be felt at the point of the noticeable thickened structure. The A1 pulley location was marked combined with relative surface landmarks and palpation techniques. While the thumb is in hyperextension position, the midline of the thumb was marked vertically to transect the MCP joint crease. The same palpation technique was performed to determine the correct position of the A1 pulley in relation to surface landmarks. The flexor pollicis longus tendon and the A1 pulley were marked.

Using the 15° stab knife, while palpating the pulley, we percutaneously released the A1 pulley in a longitudinal motion. We performed this on all fingers respectively.

Then, by a transverse incision of the palm of the hand at the head of the metacarpals, the length of the A1 pulley and the amount of pulley that was released was measured in millimeters using a caliper. We examined all the A1 pulleys for proper release and also the unwanted release of the A2 pulley, the flexor tendons for scratches, and the neurovascular tendons for any damage. The success rate of A1 pulley release was assessed using a measurement of the amount of A1 pulley that was released (millimeter), divided by the full length of the pulley that was considered as a percentage. The success rate of the release is divided into groups of 75–89%, 90–99%, and 100%.

Considering the difference of the digital nerve and arteries in the index finger and thumb, the rate of A1 pulley release and its complications were analyzed separately. Finally, data were entered into the SPSS software and statistical analysis was performed where the data were described by frequency and percentage. P value ≤ 0.05 was considered as the level of significance.

## Results

In the present study, the success rate of A1 pulley release was determined from 100 fingers of 10 cadavers including nine males and one female. Finally, the success rate of A1 pulley release was 75–89% in four fingers, followed by eleven fingers (90–99%) and eighty-five fingers (100%).

Overall, the mean of A1 pulley release for these fingers was calculated as 97.9%. Considering the different anatomy of A1 pulley in each of the fingers, the success rate of A1 pulley release in each finger was separately evaluated on the left and right hand (Table 1).

**Table 1:** Percutaneous trigger release success rate of different fingers

Variable		Success			*P* value
		75–89%	90–99%	100%	
Hand	Left	3 (6.0%)	8 (16.0%)	39 (78.0%)	0.14
	Right	1 (2.0%)	3 (6.0%)	46 (92.0%)	
Finger	Thumb	1 (5.0%)	2 (10.0%)	17 (85.0%)	0.73
	Index	2 (10.0%)	3 (15.0%)	15 (75.0%)	
	Middle	0 (0.0%)	1 (5.0%)	19 (95.0%)	
	Ring	1 (5.0%)	2 (10.0%)	17 (85.0%)	
	Little	0 (0.0%)	3 (15.0%)	17 (85.0%)	
Left hand	Thumb	1 (10.0%)	0 (0.0%)	9 (90.0%)	0.49
	Index	1 (10.0%)	3 (30.0%)	6 (60.0%)	
	Middle	0 (0.0%)	1 (10.0%)	9 (90.0%)	
	Ring	1 (10.0%)	1 (10.0%)	8 (80.0%)	
	Little	0 (0.0%)	3 (30.0%)	7 (70.0%)	
Right hand	Thumb	0 (0.0%)	2 (20.0%)	8 (80.0%)	0.29
	Index	1 (10.0%)	0 (0.0%)	9 (90.0%)	
	Middle	0 (0.0%)	0 (0.0%)	10 (100.0%)	
	Ring	0 (0.0%)	1 (10.0%)	9 (90.0%)	
	Little	0 (0.0%)	0 (0.0%)	10 (100.0%)	

As shown in Table 1, no significant difference was found in the amount of A1 pulley release in each of the fingers as compared to each other, as well as in each of the right and left hands.

Of the 10 patients evaluated in this study, overall, 4 patients showed 75–89% success rate, 11 patients showed 90–99% success, and the majority (85%) showed a complete success rate (Table 1). One cadaver was a female, and 9 cadavers were male.

In this study, superficial scrape in flexor tendons was found among 17 fingers, and 83 fingers showed no flexor tendons injuries. No vascular, digital nerve, and A2 pulley injuries were observed (Table 2).

**Table 2:** Injuries due to the procedure

Injury	Frequency
Superficial flexor tendon injury	17.0%
Pulley A2 injury	0.0%
Digital nerve injury	0.0%
Digital vascular injury	0.0%

## Discussion

The trigger finger is one of the most common causes of hand disability and a common reason for patients to be referred to an orthopedic clinic. The first line of treatment is the use of non-surgical methods such as finger rest, splinting, and corticosteroids injection that the success rate of which is reported to be 38 to 93%. When failure occurs in non-surgical treatment, the standard treatment is open-release of flexor tendon, which has a success rate of nearly 100% [21–23]. Due to the complications of open surgery, which include pain at the incision site, infection, stiffness, cross-sectional nerve incision, flexor tendon Bowstringing, sympathetic reflex dystrophy, and deformity when bending, several percutaneous techniques are currently performed with different instruments such as needle 18, pin, and blades 11 and 15 have been introduced in studies that have successful results in more than 90% of patients. So the use of these instruments has also been associated with reduced time and cost, faster recovery, and reduced pain at the incision site. However, the study of the percutaneous release using a 15° stab has not been evaluated in studies.

Due to the unique features of this stab knife, including its delicacy and its suitable length and width for surgery, the use of a 15° stab knife is less likely to damage the tendon and also less likely to cause neurovascular damage and overall less damage to the arteries and nerves, and in this regard, to evaluate this method. The present study was an experimental study to evaluate the success rate of A1 pulley release by a 15° stab knife in 100 fingers in a cadaveric model.

Our findings revealed a success rate of 75% for A1 pulley release in four fingers, followed by eleven fingers (90%) and eighty-five fingers (100%), where the mean of A1 pulley release for these fingers was found to be 97.9%. The results also showed that there was no significant difference in the amount of release between the two hands and the fingers of each hand separately. In addition, in this study, superficial scratches of flexor tendons are only in 17 fingers, and damage to the digital nerves and vessels and A2 pulley was not observed. These results demonstrate that percutaneous trigger finger release with 15° stab knife can be effectively applied as an alternative after the failure of conservative treatment. This finding is more or less in line with other available studies. Success rates of finger release have been demonstrated in the observational range from 84 to 100% by mid-term follow-up [17–19]. Studies demonstrated effective findings using different materials in a percutaneous release such as knives, scalpels, and use of ultrasound [24–27].

In a cadaveric study, a 74% success rate has been reported for percutaneous release by applying an 18-gauge needle, where A1 pulleys were found to be completely released [28]. Furthermore, 100% success rate has been achieved by an angiocath needle [29], followed by 91% up to 93% success rate via special blade with a hook to percutaneous release [26], and favorable results for knife technique in Smith’s study [30].

Percutaneous trigger finger release by using a new push knife has been addressed to be effectively applicable for providing complete release of the A1 pulley as compared to 19-gauge needle in cadaveric hands, where it provided less complication of the flexor tendon surface [31]. In the present study, percutaneous trigger finger release using a 15° stab knife revealed low complications in a cadaveric model such as superficial damage of the flexor tendon.

In 2019, a study by Kumar et al. with 43 trigger finger treatments in of 36 patients with the percutaneous method with needle 18 showed that the success rate of treatment in 81.39% of patients was appropriate and excellent. 19.61% of patients required open surgery, and their study did not show vascular or neurological complications similar to the present study [32]. Pan and colleagues in 2019 compared the release of A1 pulley with and without an ultrasound guide showed that the success rate in the group under the guidance of the ultrasound release with Hanzhang needle knife was 100%, and no side effects were observed in that group. The only operation time in the guidance of the ultrasound group was longer [33]. Compared to these studies, Jegal et al. (2018) showed that topical injection of corticosteroids after the percutaneous release can improve treatment outcomes and reduce patient pain in the short period after surgery [34].

Percutaneous release has been suggested to be a safe alternative, where trigger digit was found to be effectively managed by percutaneous release [15]. Based on the data presented in the literature, open release exhibited a low complication rate as a simple and safe procedure [35]. Open surgical release of the A1 pulley is a gold standard because of its remarkable rate of success (minimal morbidity and recurrence) [20], ranging from 60 to 97% [36–38]; nonetheless, the overall complication rates of open trigger finger release have been indicated to be between 11 and 43% [37, 39, 40]. But open trigger finger release is considered a low-risk method [41].

Percutaneous release has been introduced as a convenient and reliable procedure because of its effective outcomes [41–43], but the success rate in different studies and with different tools has been different; however, potential disadvantage has been suggested previously, where this method may be associated with damage to either nerve or tendon and recurrent triggering, depending on the type of device used for surgery [20, 28, 41, 44, 45]. In the present study, with the use of 15° stab knife due to its fineness and short length and width, only 17 flexor tendons were superficially damaged and no damage to arteries, nerves, and A2 pulley was observed. Also, due to its low width (1.6 mm), stab knife provides more maneuverability for the surgeon to be able to move in the tissue and be returned, which is not the case in blades 11 and 15. However, this is also possible in pin or needle, but because these are relatively blunt, the scratches that they create on the pulley are irregular and may deviate, while the 15° stab knife does not deviate from the left and right in the tissue and becomes more precise.

In a study (2019), patients’ satisfaction with the percutaneous method (using needle 16) and treatment with topical steroid injections was investigated. The results of their study showed that in 1 and 3 months after treatment, patients were more satisfied and the pain caused by treatment with the percutaneous method was significantly lower [46].

Will and Lubahn (2010) demonstrated the infrequent occurrence of major complications, while the rate of minor complications has been reported by authors to be remarkable and linked to wound or loss of finger range of motion [35]. Another study by Sato et al. Showed that open surgery and percutaneous release techniques can have similar effects and more appropriate than conservative therapy and corticosteroid injections (to treat and reduce recurrence) [47].

A cadaveric study indicated that similar complete A1 pulley release and injury rates have been achieved by ultrasound-assisted and percutaneous trigger finger release methods [28]. Percutaneous release of the A1 pulley using a 15 blade has been contributed to favorable efficacy and relatively good safety in a cadaveric study [48]. Moreover, incision wound-related pain is not present in the percutaneous release technique with a faster recovery rate as compared to open release [49].

Although studies emphasize on advantages of the percutaneous release procedure, many studies reported that excision of the A1 pulley was not completely performed, which can probably contribute to some complications such as the likelihood of nerve injury and longitudinal wound scar [41]. Also, the most important concern about percutaneous release is the proximity of the digital nerve to the A1 pulley, which of course, hyperextension of the finger and the use of the midpoint line for precutaneous release help to prevent the digital nerve damage [32]. In this study, the use of a 15° stab knife, in addition to its high success rate, was associated with fewer complications compared to other surgical procedures and instruments.

Finally, our finding demonstrated that treating trigger fingers via percutaneous release with a 15° stab knife is remarkably effective and relatively safe, where superficial scratch in flexor tendons was found in 17 fingers, and 83 fingers showed no flexor tendons injuries and other injuries, e.g., vascular, digital nerve, and A2 pulley injuries, which is due to the characteristics of the 15° stab knife. Some studies have also reported that using the percutaneous method in the thumb and forefinger was associated with more complications, but in the present study, using a 15° stab knife, the results were also suitable for the thumb and forefinger.

However, due to the fact that this study was performed on a cadaveric model, it seems that more extensive clinical studies using other devices as well as ultrasound-assisted can be the basis for future clinical trials. In addition, the effectiveness of treatments can be assessed by examining the time to return to normal activity in patients. Also, comparing the results of treatment with a 15° stab knife with other methods can be helpful in determining the preferred method in the treatment of the trigger finger.

One of the limitations of the present study is that we work on a cadaveric model, because they are not compatible with the living person and may be different from the clinical condition. Also, the pulley is not involved in a cadaveric model, and in clinical cases where the pulley is involved, the results may be different. Also, due to the thinness of the stab knife, it may bend under excessive pressure. Therefore, clinical studies and comparisons with other methods as well as supportive therapies are recommended.

## Data Availability

Not applicable. The datasets used and/or analyzed during the current study are available from the corresponding author on reasonable request.
